# In Patients With Parkinson’s Disease in an OFF-Medication State, Does Bilateral Electrostimulation of Tibialis Anterior Improve Anticipatory Postural Adjustments During Gait Initiation?

**DOI:** 10.3389/fnhum.2021.692651

**Published:** 2021-07-21

**Authors:** Arnaud Delafontaine, Paul Fourcade, Ahmed Zemouri, D. G. Diakhaté, Gabriel Saiydoun, Eric Yiou

**Affiliations:** ^1^CIAMS, Univ. Paris-Sud., Université Paris-Saclay, Orsay, France; ^2^CIAMS, Université d’Orléans, Orléans, France; ^3^Unisurg, Paris, France; ^4^UFR Sciences de l’Education de la Formation et du Sport, Gaston Berger University, Saint-Louis, Senegal; ^5^Department of Cardiac Surgery, Henri Mondor University Hospital, APHP, Créteil, France; ^6^University of Paris-Est Creteil, UFR Médecine-Pharmacie, Créteil, France; ^7^Institut Mondor de Recherche Biomedicale, IMRB, Inserm U955, Faculté de Santé de Créteil, Creteil, France

**Keywords:** Parkinson’s disease, functional electrical stimulation, gait initiation, anticipatory postural adjustments, tibialis anterior

## Abstract

A complete lack of bilateral activation of tibialis anterior (TA) during gait initiation (GI), along with bradykinetic anticipatory postural adjustments (APAs), often occurs in patients with Parkinson’s disease (PD) in their OFF-medication state. Functional electrical stimulation (FES) is a non-pharmacological method frequently used in neurorehabilitation to optimize the effect of L-DOPA on locomotor function in this population. The present study tested the potential of bilateral application of FES on TA to improve GI in PD patients. Fourteen PD patients (OFF-medication state, Hoehn and Yahr state 2-3) participated in this study. They performed series of 10 GI trials on a force-plate under the following experimental conditions: (1) GI without FES (control group), (2) GI with 2Hz-FES (considered as a very low FES frequency condition without biomechanical effect; placebo group) and (3) GI with 40Hz-FES (test group). In (2) and (3), FES was applied bilaterally to the TA during APAs (300 mA intensity/300 μs pulse width). Main results showed that the peak of anticipatory backward center of pressure shift, the forward center of mass (COM) velocity and shift at foot off were significantly larger in the 40 Hz FES condition than in the control condition, while the duration of step execution was significantly shorter. In contrast, the capacity of participants to brake the fall of their COM remained unchanged across conditions. Globally taken, these results suggest that acute application of 40-Hz FES to the TA may improve the capacity of PD patients to generate APAs during GI, without altering their balance capacity. Future studies are required before considering that TA FES application might be a valuable tool to improve GI in PD patients and be relevant to optimize the effects of L-DOPA medication on locomotor function.

## Introduction

Gait initiation (GI), the transient period between steady-standing posture and steady-state walking, is a functional task known to be altered in patients with Parkinson’s Disease (PD) (Halliday et al., 1998). Alteration in GI has been identified as a source of fall in neurological patients (Stolze et al., 2004). GI is classically decomposed into three successive phases: the postural phase, which precedes the swing heel-off (and corresponding to the so-called “anticipatory postural adjustments,” APAs), the unloading phase (from swing heel-off to toe-off) and the execution phase ending at the time of foot-contact. During APAs, the tibialis anterior (TA) are activated bilaterally, which is responsible of backward center-of-pressure (COP) shift. This COP shift is necessary to generate the initial forces to propel the center-of-mass (COM) forward during the execution phase, and reach the desired step length and velocity (Lepers and Breničre, 1995). During step execution, the COM falls under gravity effect, and this fall is actively braked by the activation of stance leg triceps surae, which acts to attenuate the impact of the swing limb at foot contact (Honeine et al., 2013).

A complete lack of bilateral TA activation, along with bradykinetic APAs often occurs in PD patients in their OFF-medication state (Halliday et al., 1998), which may be one of the factors responsible for the symptoms of “start hesitation” typical of this population (Delval et al., 2014). Altered active COM braking has also been reported in patients with severe PD patients (Chastan et al., 2009).

Classical treatment of PD includes L-DOPA administration (Palmisano et al., 2020). It is however noteworthy that the positive effects of this pharmacological treatment is controversial (Rocchi et al., 2002). L-DOPA may indeed induce dyskinesia (Curtze et al., 2015), which may alter balance control in PD patients in their ON-medication state when compared to their OFF-medication state (Armand et al., 2009).

Recent studies reported that Functional Electrical Stimulation (FES), a non-pharmacological rehabilitation method, may be relevant to optimize the effect of L-DOPA on locomotor function. FES is a means of producing an active muscle contraction controlled in such a way to provide functional movement to assist everyday tasks. Sijobert et al. (2016) showed that plantar sensitive electrical stimulation during steady-state walking reduced freezing by about 12% in PD patients. Mann et al. (2008) reported that the application of FES on the common peroneal nerve of PD patients increased the average stride length, increased the distance covered during a 3-min walk test, and decreased the risks of falls in this population. FES stimulation might also specifically ameliorate gait in patients with freezing of gait (FOG) (Djurić-Jovičić et al., 2013). However, it is noteworthy that Mancini and Horak (2010) stressed that the greatest limitation of these clinical approaches to rating balance is that they cannot specify what type of balance problem a subject suffers in order to direct a treatment.

To our knowledge, no studies to date investigated whether FES may be efficient to facilitate GI in PD patients. The present study thus tested the potential of bilateral application of FES on TA to improve GI in PD patients.

## Methods

Fourteen PD patients [11 men and 3 women, aged 69 ± 7 years, height 166 ± 8.2 cm, body-mass 66 ± 15 kg [(mean ± SD)]; see Table 1, classified Hoehn and Yahr states 2-3 were included in the experiment. Only PD patients in their OFF-medication state (i.e., after 12-h withdrawal from their antiparkinsonian’s L-DOPA medications) were included because this is the condition where alterations in APAs development and associated TA activity mainly occur. All patients reported FOG. Exclusion criteria included: visual, hearing or orthopedic problems, other identified neurological troubles, dementia, severe dyskinesia, a score <25 on the Mini Mental State Exam, implanted deep brain stimulator and response fluctuations. The patients had no medical history of falling. All subjects gave informed written consent as required by the Declaration of Helsinki. The experiment was approved by the “Comité de Protection des Personnes Ile-de-France XI” under identification number 19028-60429.

**TABLE 1:** Demographic characteristics and clinical parameters of PD patients.

**PD patients**	**Sex**	**Age (years)**	**Height (cm)**	**Body-mass (kg)**	**Disease duration (years)**	**Hoehn and Yahr stage**	**MDS-UPDRS motor examination (section III)**	**STS (numbers/15 s)**	**MMSE (points)**
Subject 1	Male	63	158	62	4	3	28	8	30
Subject 2	Male	74	174	70	10	2	25	8	30
Subject 3	Male	80	170	62	5	3	24	4	25
Subject 4	Female	75	169	76	12	2	30	8	30
Subject 5	Male	70	170	76	8	2	19	11	30
Subject 6	Male	64	170	70	15	3	32	7	25
Subject 7	Female	62	158	45	12	2	19	8	30
Subject 8	Male	78	173	82	4	3	24	5	27
Subject 9	Male	70	176	87	6	3	28	6	27
Subject 10	Male	71	160	66	8	2	27	7	27
Subject 11	Male	68	160	52	7	2	19	9	29
Subject 12	Male	57	155	48	5	2	35	7	28
Subject 13	Male	63	157	40	8	3	21	8	30
Subject 14	Female	71	180	90	6	2	32	5	28
Mean (SD)	–	69 (7)	166 (8.2)	66 (15)	8 (3)	2 (0.5)	26 (5.3)	7 (2)	28 (2)

*MDS-UPDRS, Movement disorders society-Unified Parkinson’s Disease Rating Scale; STS, Sit-to-Stand Task, STS score corresponds to the number of rising from a chair performed during 15 s; MMSE, Mini Mental State Exam.*

MDS-UPDRS: Movement disorders society-Unified Parkinson’s Disease Rating Scale; STS: Sit-to-Stand Task. STS score corresponds to the number of rising from a chair performed during 15 seconds; MMSE: Mini Mental State Exam.

Each participant performed series of GI trials at a spontaneous velocity on a force-plate (0.9 × 1.80 m, AMTI, Watertown, United States) following an acoustic signal delivered by the experimenter. Ten GI trials were performed in each of the three following experimental conditions (for a total of *N* = 30 trials): (1) GI without FES (control), (2) GI with 2 Hz FES (considered as a very low FES frequency condition without biomechanical effect), and (3) GI with 40 Hz FES. In (2) and (3), FES (referee Compex Wireless Professional^®^) was applied bilaterally to TA during APAs with an intensity of 300 mA and a pulse width of 300 μs. The order of the experimental conditions was randomly assigned across participants to avoid rank effects.

Force-plate data were low-pass filtered using a second order Butterworth filter with a 10 Hz cut-off frequency (Winter, 1990; Sinclair et al., 2013). The COP anteroposterior coordinate was computed from force-plate data. The instantaneous COM acceleration was determined from the ground reaction forces. COM velocity and position were computed with successive numerical integrations of COM acceleration (Yiou et al., 2016). Data were collected at a rate of 500 Hz. Data acquisition was controlled by a custom-made program written in MATLABTM [Version 5.3 (R11), The MathWorks Inc., United States].

**FIGURE 1:**
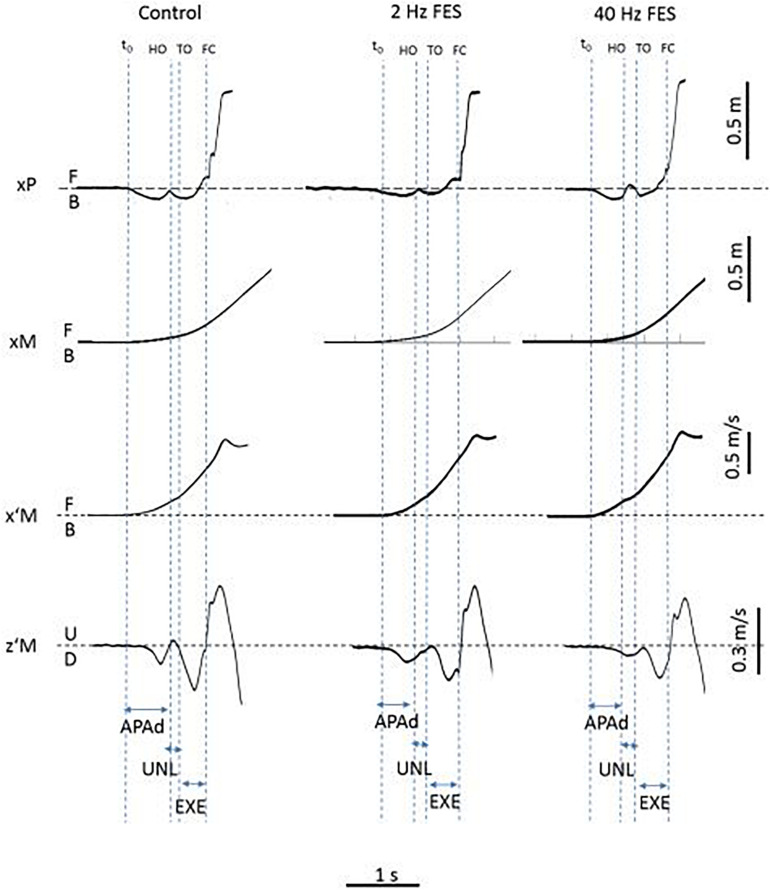
Typical biomechanical traces of gait initiation obtained in the three experimental conditions (Control, 2 and 40 Hz FES) and showing each dependent variable (one trial in one representative PD patient). *Biomechanical traces*. xP, xM, x’M, z’M: cenete of pressure (COP) displacement, center of mass (COM) displacement and COM velocity along the anteroposterior (x) and vertical (z) axis, respectively. B, F, U, D: backward, forward, upward and downward direction, respectively. t0, HO, TO, FC: onset of biomechanical traces, swing heel-off, toe-off and foot-contact, respectively. *Experimental variables*. APAd, UNL, EXE: time-windows for APAs, unloading and execution phases of gait initiation.

Classical GI biomechanical parameters were analyzed. Temporal parameters included: duration of APAs (APAd, from onset variations of biomechanical traces to heel-off), unloading (UNL, from heel-off to toe-off) and execution phase (EXE, from toe-off to heel-contact). Spatial parameters included: peak backward COP shift (xP_MAX_), forward COM velocity (x’M_TO_) and COM position at toe-off (xM_TO_), forward COM velocity at foot-contact (x’M_*FC*_), step length (SL) and Braking Index (BI). GI biomechanical traces obtained in one representative PD subject and showing temporal parameters are reported in Figure 1.

Mean values and standard deviations were calculated for each dependent variable. Repeated-measures ANOVAs were used to test the effect of the condition (control, 2 Hz FES, 40 Hz FES) on each variable after having checked normal distribution with the Shapiro-Wilk test. A significant outcome was followed up with the Tukey *post hoc* test. The threshold of significance was set at *p* < 0.05. The Cohen’s d (d’) was used to assess effect sizes. Effect sizes were classified as trivial (<0.2), small (0.2–0.49), medium (0.5–0.79), and large (≥0.8).

## Results

**FIGURE 2:**
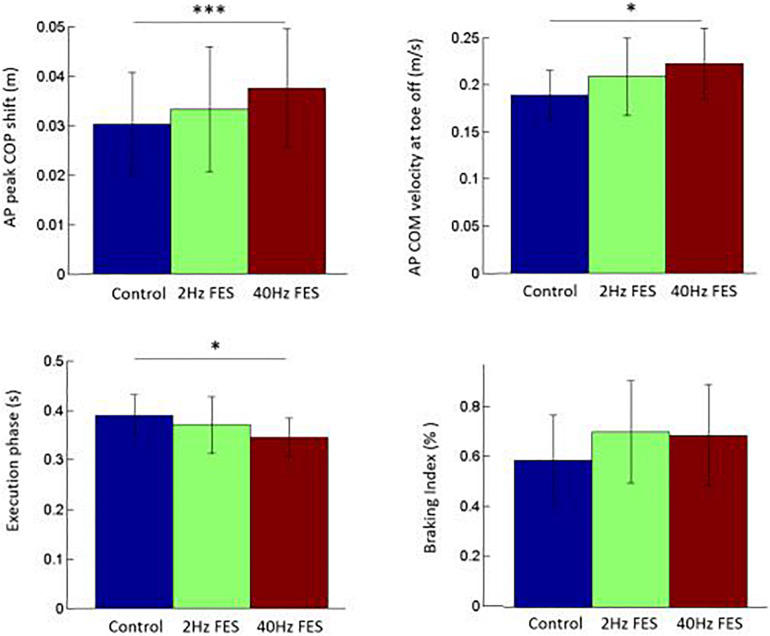
Comparison of selected gait initiation parameters between the three conditions. Reported are mean values (all participants together) ± 1 SD. AP: anteroposterior direction. COM, COP: center of mass, center of pressure, respectively. ^∗^, ^∗∗∗^ indicates a significant difference between bars with *p* < 0.05 and *p* < 0.001 as revealed with the *post hoc* analysis.

Statistical analysis showed that there was a significant effect of the condition on the following variables (Figure 2): xP_MAX_ [*F*(2, 26) = 8.41, *p* < 0.01], x’M_TO_ [*F*(2, 26) = 7.80, *p* < 0.01], xM_TO_ [*F*(2, 26) = 13.64, *p* < 0.001] and EXE [*F*(2, 26) = 4.15, *p* < 0.05]. Specifically, *post hoc* test showed that xP_MAX_ (*p* < 0.001; *d*′ = 0.38), x’M_TO_ (*p* < 0.05; *d*′ = 0.58) and xM_TO_ (*p* < 0.001; *d*′ = 0.17) were significantly larger in the 40 Hz FES condition than in the control condition, while EXE was significantly shorter (*p* < 0.05, *d*′ = 0.62, Figure 2). There was no significant effect of the condition on the following variables: APAd (590 ± 26 ms; mean of average values and standard deviation (SD) obtained in the three conditions together), UNL (163 ± 6 ms), SL (56 ± 1 cm), x’M_*FC*_ (0.80 ± 0.01 m/s) and BI (65 ± 6%).

## Discussion

When gait is initiated at spontaneous velocity as in the present study, PD patients are known to develop APAs of lower amplitude than healthy age-matched participants, i.e., their APAs are hypometric (Halliday et al., 1998; Palmisano et al., 2020). The present study thus tested the potential of bilateral application of FES on TA to improve GI in PD patients. The finding that, in the 40 Hz FES condition, PD patients spontaneously initiated gait with a larger APAs amplitude (in terms of peak backward COP shift and forward COM velocity/displacement at foot-off) than in the control condition without modifying their APAs duration, suggests that this symptom was attenuated by the treatment. As APAs amplitude is related to TA activation (Lepers and Breničre, 1995), this positive effect of FES can be ascribed to a putative increased in TA activation provided by the FES (not recorded). Furthermore, the duration of step execution was significantly shorter in the 40 Hz condition than in the control condition, while step length remained unchanged. In accordance with the literature (Lepers and Breničre, 1995), this quicker step execution can likely be ascribed, at least partly, to the increased APAs amplitude.

These results are in agreement with two previous clinical studies focusing on the effects of ankle dorsi-flexors assistance provided by 40 Hz FES application to lower legs on gait in PD patients (Mann et al., 2008; Popa and Taylor, 2015). For example, in the feasibility study of Mann et al. (2008), seven PD patients who exhibited freezing in gait used an FES device for a period of 8 weeks. This study showed that the FES treatment improved some gait parameters over the tested period of use with a carryover effect that is maintained without stimulation during that time and an immediate reduction in the frequency of falls. An immediate effect of FES was demonstrated over a 3-min walk but not over a 20-m walk. Furthermore, these authors showed that these improvements in gait persisted on reassessment 4 weeks after FES withdrawal, although the frequency of falls returned to pretreatment levels. Similar beneficial effects of FES application on gait were also reported in the pilot study of Popa and Taylor (2015) involving 11 PD patients. These authors showed that the mean walking speed, step length, and cadence of gait increased following a 2 weeks lower legs FES application. These authors further showed that FES treatment applied to the upper limb was also efficient to improve manual speed and dexterity. Interestingly, these motor improvements were associated with improvements in health related quality of life. These preliminary results thus suggest that FES application might be useful to attenuate both hypokinesia and bradykinesia in PD patients. However, it is clear that further studies involving a larger number of participants are required to confirm (or not) these beneficial effects of FES on the motor behavior of PD patients.

In the present study, it is noteworthy that, for the GI velocity spontaneously adopted by the PD patients, balance control was not altered by the FES since the active COM braking remained unchanged across conditions. COM braking is due to the activation of the stance leg triceps surae (TS) during the GI execution phase (Honeine et al., 2013). It therefore seems that the changes in TA activation with the 40 Hz FES application did not alter the TS antagonistic activity with a reciprocal inhibition effect from TA to TS. This finding was important to stress since the COM braking during GI is a main issue in PD patients (Chastan et al., 2009) and should not be further degraded by the treatment. Note that it was not possible to properly analyze EMG of lower leg muscles due to artifacts associated with FES application.

When compared to previous works in the literature on healthy elderly initiating gait at a spontaneous velocity (Halliday et al., 1998; Chastan et al., 2009; Palmisano et al., 2020), it seems that the GI parameters obtained by PD patients in the control condition were not dramatically altered. Note, however, that the peak backward COP shift (2.96 ± 1.02 cm) and the forward COM velocity at foot-off (0.18 ± 0.02 m/s) were both lower than values reported in healthy elderly (e.g., 3.54 ± 1.40 cm and 0.50 ± 0.13 m/s, respectively, in Halliday et al. (1998)). Following the 40 Hz FES application, the peak backward COP shift reached (3.67 ± 1.2 cm), a value which might be considered as within a normal range. The forward COM velocity at foot-off also increased (0.21 ± 0.03 m/s) but still remained below a normal range. In other words, it seems that acute application of 40 Hz FES failed to induce a complete recovery of APAs in PD patients, a finding that contrasts with the effects of L-dopa intake on the same APAs parameters as in the present study (Palmisano et al., 2020).

The present study adds to the growing efforts made by researchers to improve APA associated with GI in PD patients by the means of non-pharmacological/non-invasive devices such as powered ankle orthosis (Petrucci et al., 2019), self-triggered stimulus lowering stance side support surface, vibrations applied beneath the stance-side support surface (Creath et al., 2013), whole-body vibration (Fischer et al., 2019), lateral pull applied to the pelvis by motor-driven robotic system (Mille et al., 2007) or rhythmic auditory stimulus (Ghai et al., 2018). Study from our laboratory (not published) further suggests that acute ankle stretching may also be efficient to reduce ankle stiffness and improve APA in this population (see Vialleron et al., 2020 for a review on the effect of stretching on gait). Because gait and balance problems often respond poorly to treatment with anti-parkinsonian medications, and to other interventions such as deep brain stimulation (Bonnet et al., 1987; Krack et al., 2003), it is admitted that physical therapy interventions are an important clinical treatment for individuals with PD. These non-pharmacological methods might potentially be integrated in physical training protocols and/or be used by PD patients to provide assistance during locomotor tasks such as gait initiation and/or steady-state gait. We acknowledge that this preliminary study has several limitations, among which: (1) the PD patients involved in the experiment had relatively low-level of motor impairment. It is not known whether acute TA FES application may also be beneficial to PD patients with more severe symptoms; (2) the sample size was relatively small (*n* = 14 participants). It follows that the results may not be generalizable to all PD participants; (3) only acute TA FES effects were considered. To what extent PD patients might beneficiate from long-term TA FES application is unknown; (4) the carry-over effects were not evaluated.

Despite these limitations, the results of the present preliminary study along with studies of the literature (Mann et al., 2008; Popa and Taylor, 2015; see also Sujith, 2008 for a review on FES on neurological disorders) are encouraging. Future studies should address these limitations before considering that TA FES application might be a valuable tool to improve GI in PD patients and be relevant to optimize the effects of L-DOPA medication on locomotor function.

## Data Availability Statement

The raw data supporting the conclusions of this article will be made available by the authors, without undue reservation.

## Ethics Statement

The studies involving human participants were reviewed and approved by Comité de Protection des Personnes Ile-de-France XI under identification number 19028-60429. The patients/participants provided their written informed consent to participate in this study.

## Author Contributions

AD and EY wrote the manuscript. PF performed the statistical analysis. EY, DD, AZ, and GS reviewed the manuscript. All authors contributed to the article and approved the submitted version.

## Conflict of Interest

The authors declare that the research was conducted in the absence of any commercial or financial relationships that could be construed as a potential conflict of interest.
